# Correction: Two new species of the *Entoloma quadratum–murrayi* complex in Japan

**DOI:** 10.1371/journal.pone.0326181

**Published:** 2025-06-10

**Authors:** The PLOS ONE Staff

## Notice of Republication

This article was republished on May 23, 2025, to rectify an error in the title introduced during the typesetting process. The publisher apologizes for this error. Please download this article again to view the correct version. The originally published, uncorrected article and the republished, corrected articles are provided here for reference.

## Supporting information

S1 File (PDF)

S2 File (PDF)
